# Computational approaches for molecular characterization and structure-based functional elucidation of a hypothetical protein from *Mycobacterium tuberculosis*

**DOI:** 10.5808/gi.23001

**Published:** 2023-06-30

**Authors:** Abu Saim Mohammad Saikat

**Affiliations:** Department of Biochemistry and Molecular Biology, Life Science Faculty, Bangabandhu Sheikh Mujibur Rahman Science and Technology University, Gopalganj 8100, Bangladesh

**Keywords:** *Mycobacterium tuberculosis*, protein function prediction, protein-protein interactions, protein structure prediction, Ramachandran plot

## Abstract

Adaptation of infections and hosts has resulted in several metabolic mechanisms adopted by intracellular pathogens to combat the defense responses and the lack of fuel during infection. Human tuberculosis caused by *Mycobacterium tuberculosis* (MTB) is the world’s first cause of mortality tied to a single disease. This study aims to characterize and anticipate potential antigen characteristics for promising vaccine candidates for the hypothetical protein of MTB through computational strategies. The protein is associated with the catalyzation of dithiol oxidation and/or disulfide reduction because of the protein’s anticipated disulfide oxidoreductase properties. This investigation analyzed the protein's physicochemical characteristics, protein-protein interactions, subcellular locations, anticipated active sites, secondary and tertiary structures, allergenicity, antigenicity, and toxicity properties. The protein has significant active amino acid residues with no allergenicity, elevated antigenicity, and no toxicity.

## Introduction

Pathogenic mycobacteria are significant sources of illness in humans and animals. Despite the accessibility of antibiotics and chemotherapeutic candidates that are efficient against some mycobacteria, the emergence of drug-resistant strains necessitates the development of new active molecules and intervention strategies [1-3]. Within more than 130 years since discovering *Mycobacterium tuberculosis* (MTB) as the causative microorganism responsible for human tuberculosis (TB) by Robert Koch, numerous scientific advances have been made to help cope with this significant pathogen. However, despite this progress, MTB still holds many unresolved secrets. Much work remains to be done to translate the essential findings from recent research into novel strategies against the pathogen [4-7]. Tuberculosis, one of the ancient recorded human diseases, continues to be one of the leading causes of death, claiming two million lives annually. TB affects bone, the central nervous system, and many other physiological systems. However, it is essentially a pulmonary illness caused by the precipitation of aerosolized MTB onto lung alveolar surfaces. From this point, the causes of the illness are contingent on the immunological reactivity of the host to varying degrees [8-10].

MTB has an irregular, highly recurrent life cycle that encompasses a varied and heterogeneous spectrum of habitats and physiologic states, many of which are unique from other infections. It is not unanticipated that MTB has conceived a specific set of metabolic capacities to facilitate its adaption to and movement across hosts, given its peculiar, if not distinctive, environment [11-13].

Numerous advancements in computational biology have made it possible to construct diverse technologies and strategies for predicting protein structure and the identification of sequence commonalities for active investigation and the analysis of active site residue relationships [14-16]. A bioinformatics examination of the proteins enables one to assess their three-dimensional architectural structure, categorize novel features, investigate specific processes to understand our biological lineage, uncover different clusters, and assign the proteins’ function. The obtained information can also convey reasonable pharmacological strategies and assets in developing promising anti-disease medications [17-19]. The hypothetical protein from MTB consists of disulfide oxidoreductase involved in the catalyzation of dithiol oxidation and/or disulfide reduction of target sites in MTB.

## Methods

### Sequence retrieval

The amino acid sequence of the protein was obtained from the NCBI database in FASTA format. The physicochemical properties were determined using ProtParam (ExPASy) and SMS v.2.0 programs. Afterwards, the subcellular location of the selected protein was determined. This study also anticipated the protein family, superfamily, domain, coil, and folding pattern of the protein. The STRING program was used for protein protein interaction determination. Moreover, secondary structural documentation was performed using the SOPMA, DISOPRED (v. 3.0), and SPIPRED (v. 4.0) programs. The tertiary structure was predicted using the Modeller program with the HHpred database and validated by the PROCHECK, Verify3D, and ProSA-web tools. Furthermore, the CASTp server was used for active site determination of the selected protein present in MTB. Additionally, the antigenicity, allergenicity, and toxicity properties of the protein were determined.

### Physicochemical properties

The physicochemical parameters of the protein were evaluated by the ProtParam assessment tool of the ExPASy server program [20] and the SMS v.2.0 program (https://www.bioinformatics.org/sms2/index.html, accessed on September 10, 2022).

### Subcellular localization identification

The CELLO (v.2.5) [21,22], PSORTb (v3.0) [23], HMMTOP (v.2.0) [24,25], and TMHMM (v.2.0) [26,27] programs are used to detect the subcellular localization and protein topology analysis.

### Prediction of the protein family, superfamily, domain, coil, and folding pattern

The NCBI CD tool was used to anticipate the conserved domain [28]. The GenomeNet [29], Pfam program [30], SuperFamily program [31], and ScanProsite tool [32] used for the evolutionary relationships determination.

### Protein-protein interaction

The STRING program (v.11.5) [33] was used to determine the protein-protein (pr-pr) interaction.

### Secondary structural assessment

The SOPMA program was used following the default parameters similarity threshold (8), window width (17), and the number of states (4) [34]. DISOPRED (v.3.0) [35] and the SPIPRED (v.4.0) [36] used for the determination of further secondary characteristics and protein topology.

### Structure prediction and validation

The tertiary structure of the protein is generated by using the Modeller program [37]. The HHpred tool selected the most suitable template for protein structure anticipation [38-40]. The PROCHECK and Verify3D programs of the SAVES (v.6.0) tool were used for the structural validation of the protein [41]. Additionally, the ProSA-web program was used to calculate the Z-score and validate the modeled 3D structure of the protein [42].

### Active sites determination

The CASTp program was used to determine the active sites in the protein [43].

### Antigenicity, allergenicity, and toxicity

The VaxiJen (v2.0) program [44] was used to determine the protein’s antigenicity. Moreover, the AllerTOP (v. 2.0) program was used to predict the allergenicity of the protein [45]. The ToxinPred program [46] was used to demonstrate the toxicity of the protein.

## Results and Discussion

### Sequence retrieval

The protein’s amino acid sequence was retrieved from the NCBI database in FASTA format. The protein contains 173 amino acids (Table 1).

### Physicochemical parameters determination

**Fig. 1.**
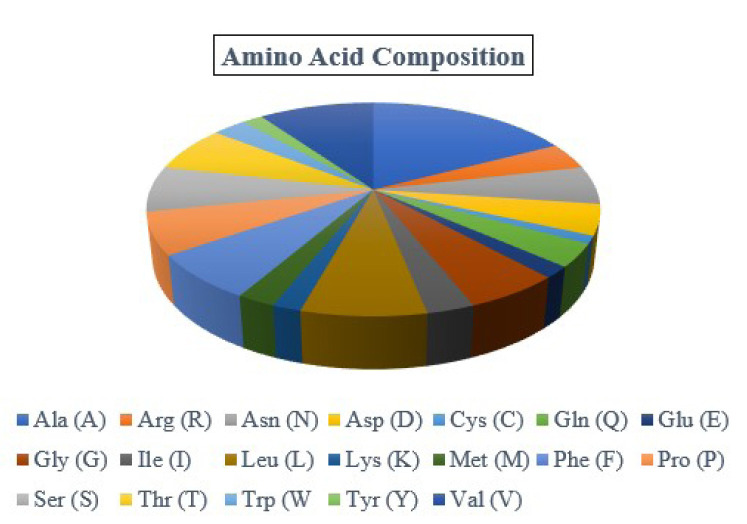
Amino acid composition. The protein contains Ala (30, 17.3%), Arg (7, 4.0%), Asn (10, 5.8%), Asp (8, 4.6%), Cys (2, 1.2%), Gln (6, 3.5%), Glu (3, 1.7%), Gly (10, 5.8%), Ile (5, 2.9%), Leu (13, 7.5%), Lys (3, 1.7%), Met (4, 2.3%), Phe (12, 6.9%), Pro (11, 6.4%), Ser (12, 6.9%), Thr (12, 6.9%), Trp (5, 2.9%), Tyr (3, 1.7%), and Val (17, 9.8%).

By examining the properties of each amino acid in the protein, it is possible to comprehend how its physicochemical properties were characterized. The ProtParam program estimated the physicochemical characteristics. The protein comprises 173 amino acids, whereas Ala (n = 30, 17.3%) is the most abundant amino acid in the protein sequence (Table 2, Figure 1). There is no His (H) in the protein sequence. The half-life of a protein is defined as the time required for the radio-labeled focal protein concentration to fall by 50% relative to the quantity at the beginning of the chasing [47]. The estimated half-life for the protein of about 30 h (mammalian reticulocytes, *in vitro*), >20 h (yeast, *in vivo*), and >10 h (*Escherichia coli*, *in vivo*). The demonstrated isoelectric point (pI), the total number of atoms, and molecular weight as of 5.19, (4.98*), 2,572, and 18,382.98 Dalton (Table 2).

Moreover, the total number of positively (Arg + Lys) and negatively charged residues (Asp + Glu) are 10 and 11 in the protein. The instability index (29.40) demonstrates protein stability, whereas the aliphatic index (86.42) denotes protein balance over a broad temperature scale. The grand average of hydropathicity (GRAVY, 0.334) determines the enhancement of thermostability [48].

### Subcellular location identification and protein topology prediction

The computerized estimation of the subcellular location of bacterial proteins is essential for proteome categorization and for selecting novel therapeutic targets and vaccination candidates. Various subcellular localization predictors have been created in recent years, including both generic localized and feature-based predictors [49-52]. The CELLO (v.2.5) and PSORTb (v3.0) predicted the subcellular location of the protein as extracellular (Table 3).

Moreover, transmembrane helix identification in integral membrane proteins is an essential bioinformatics component. In addition to predicting individual transmembrane helices, the most effective approaches to date strive to predict the complete topology of the protein, including the entire count of transmembrane helices and their direction related to the membrane [27,53]. The TMHMM (v.2.0) and HMMTOP (v.2.0) programs anticipated the protein has a single transmembrane helix (at 12–30 region in the protein residue). Most membrane proteins’ transmembrane portions have helices as their secondary structures. When a membrane protein is conducting its task, whether sending messages throughout the membrane or assisting an ion channel in unlocking or closing, the transmembrane helices frequently move together [54-57].

### Prediction of the protein family, superfamily, domain, coil, and folding pattern

The Conserved Domain Database (CDD) intends to annotate biomolecular sequences with the evolutionarily conserved protein domain placement. A repository of pre-computed domain identification is kept for NCBI’s Entrez database-tracked proteins, and real-time search facilities are provided. CDD also facilitates comparative analysis of protein families employing conserved domain architectures, and a new curation effort focuses on giving functional categorization of various subfamily structures [58-60]. The CDD tool classified the protein as protein disulfide oxidoreductase (domain architecture ID 10122406, accession ID cd03011) associated with the catalyzation of dithiol oxidation or disulfide reduction of target proteins [61].

The GenomeNet program identified six different motifs, including AhpC-TSA (position between 43-144, independent E-value 6.8 × 10^-15^), Redoxin (position between 44–137, independent E-value 1.60 × 10^-10^), thioredoxin (position between 52–114, independent E-value 1.0 × 10^-4^), thioredoxin-2 (position between 59–164, independent E-value 4.30 × 10^-5^), thioredoxin-8 (position between 61-146, independent E-value 3.6 × 10^-4^), and thioredoxin-9 (position between 57-108, independent E-value 8.3 × 10^-2^). The Pfam program [62] and the ScanProsite tool also validated the six motifs, including AhpC-TSA (accession ID PF00578), Redoxin (accession ID PF08534), thioredoxin (accession ID PF00085), thioredoxin-2 (accession ID PF13098), thioredoxin-8 (accession ID PF13905), and thioredoxin-9 (accession ID PF14595). Moreover, the SuperFamily program anticipated the protein as a member of the thioredoxin-like superfamily (accession ID 52833, E-value 2.1 × 10^-30^). Thioredoxins are small proteins composed of around one hundred amino acid residues that participate in numerous redox processes. Thioredoxins operate by reversibly oxidizing an active center disulfide bond. An intramolecular disulfide bond connects the two cysteine residues in reduced or oxidized forms [63-68].

### Protein-protein interaction

**Fig. 2.**
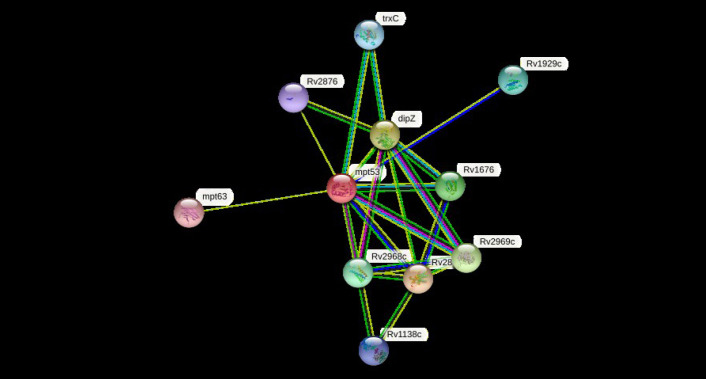
The STRING network determines the protein-protein (pr-pr) interactions. For node content: colored nodes–query proteins and first shell of interactors, white nodes–the second shell of interactors, empty nodes–proteins of unknown 3D structure, filled nodes–some 3D structure is known or predicted.

The cellular machinery is supported by proteins as well as their functional connections. For a comprehensive comprehension of biological events, their connection network must be considered, yet the existing knowledge on protein-protein relationships is inadequate and of different annotation granularity and trustworthiness. The STRING database attempts to gather, assess, and integrate all publicly accessible sources of knowledge on protein-protein interactions and supplement them with computational predictions. It seeks to establish a worldwide network that is complete and objective, incorporating both primary (physical) and secondary (functional) linkages [69-72]. The STRING program (v.11.5) was performed to determine the protein-protein (pr-pr) interaction (Fig. 2). The string program demonstrated the functional fellows with the scores as of trxC (0.795), Rv2876 (0.734), dipZ (0.884), Rv1929c (Rv1929c), mpt63 (0.731), Rv1676 (0.818), Rv2968c (0.798), Rv1929c (0.795), Rv2969c (Rv2969c), and Rv1138c (0.763).

The trxC, Rv2876, dipZ, Rv1929c, mpt63, Rv1676, Rv2968c, Rv1929c, Rv2969c, and Rv1138c are the thioredoxin that participates in multiple redox reactions through the reversible oxidation of its active center dithiol to a disulfide and catalyzes dithiol-disulfide exchange reactions, possible conserved transmembrane protein, cytochrome c-type biogenesis protein, conserved hypothetical protein, immunogenic protein mpt63 (antigen mpt63/mpb63), uncharacterized protein, probable conserved integral membrane protein, conserved hypothetical protein (uncharacterized), possible conserved membrane or exported protein, and possible conserved membrane or exported protein, respectively [73-76]. The mtp53 is a soluble secreted antigen mpt53 precursor, disulfide oxidoreductase, that speeds up the oxidation of diminished, unfolded secreted proteins to make disulfide bonds [77].

### Secondary structural assessment

**Fig. 3.**
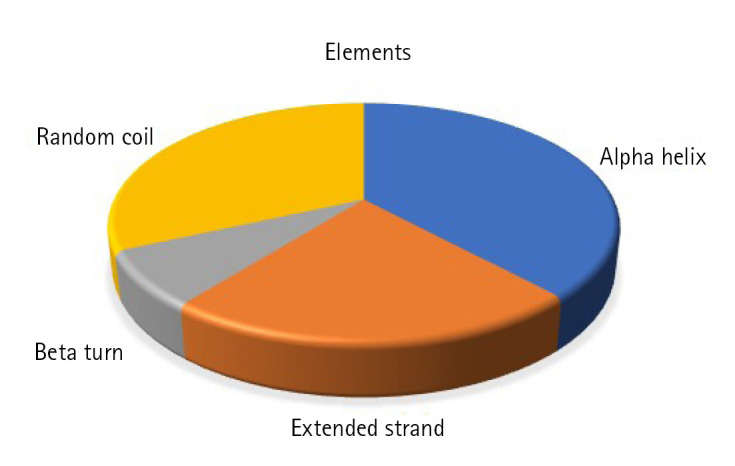
Secondary structural elements. The alpha helix (n = 66, 38.15%), extended strand (n = 39, 22.54%), beta-turn (n = 13, 7.52%), and random coil (n = 55, 31.79%).

Static high-resolution structures have contributed significantly to our protein structure and molecular activity knowledge. As structural biology has progressed, it has become evident that high-resolution structures alone cannot adequately represent the molecular basis for the structure and the action of proteins in solution [78-80]. The secondary structural components, such as helix, sheet, coil, and turn, strongly correlate with proteins' operation, architecture, and interaction [81-84]. The SOPMA program identified the alpha-helix (n = 66, 38.15%), extended strand (n = 39, 22.54%), beta-turn (n = 13, 7.52%), and random coil (n = 55, 31.79%) (Fig. 3).

**Fig. 4.**
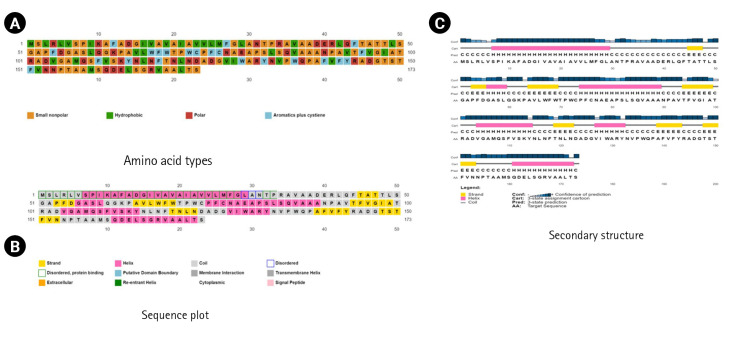
The secondary structural inquiry and assessments. (A) The amino acid types. (B) Sequence plot. (C) Secondary structure.

The SPIPRED (v.4.0) and the DISOPRED (v.3.0) programs were used to determine the secondary structure, sequence plot, and transmembrane topology (Fig. 4).

### Structure prediction and validation

Homology modeling is a technique for constructing the three-dimensional structures of proteins based on their primary sequence and using existing information from structural matches to other proteins. Sequence/structure compatibility is improved in the homology modeling procedure, a framework is constructed, and side chains are appended [85,86]. The HHpred is an accessible, collaborative web service for protein bioinformatics analysis. Experts and non-experts have access to a vast array of integrated outside-generated, cutting-edge bioinformatics tools [87].

The HHpred is a robust technology for remote homology determination and structure prediction, first constructed as hidden Markov models as well as popularized by the first pairwise comparison study of homologous protein patterns. It permits several repositories, such as PDB, CDD, Pfam, SMART, SCOP, and COG [38]. It accepts a single query array or many lineups as an entry and provides the results via a user-friendly layout similar to PSI-BLAST. Local or worldwide integration and the discovery of secondary systems are among the screening capabilities. HHpred can construct multiple inquiry prototypes, various model alignments with numerous schemes, and three-dimensional representations calculated with the Modeller program from these combinations [39]. The most suitable template (HHpred ID: 1LU4_A, PDB ID: 1LU4) was selected with the probability (99.92%), E-value 1.7 × 10^-22^, and target length of 136.

**Fig. 5.**
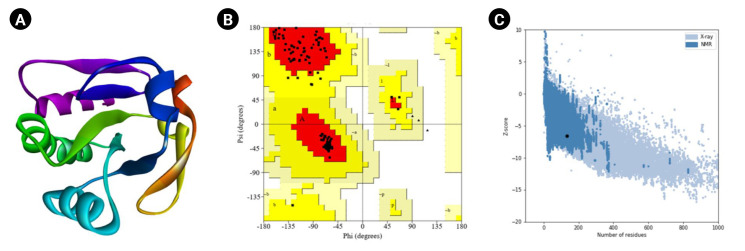
Tertiary structural assessment. (A) The 3D structure anticipated by Modeller. (B) The 3D structural assessment by Ramachandran plot statistics obtained from the SAVES program. The residues in most favored regions (n = 106, 91.4%), residues in additional allowed regions (n = 10, 8.6%), the number of glycine residues (shown as triangles) is 8, and the number of proline residues is 9. (C) The Z-score (–6.53) to assess the 3D structure.

Moreover, the PROCHECK program of the SAVES (v.6.0) tool was used for the Ramachandran plot assessment (Fig. 5). The amino acid sequences in the most favored regions, residues in additional allowed regions, the number of glycine residues (shown as triangles), and the number of proline residues is 9 are 106 (91.4%), 10 (8.6%), 8, and 9, respectively. Likewise, the Verify3D program demonstrated as 100% of the residues averaged a 3D-1D score (≥0.2), whereas at least 80% of the amino acids scored (≥0.2) in the 3D/1D profile to pass [88]. Identifying flaws in theoretical and experimental representations of protein architecture is a fundamental challenge in structural biology. ProSA is a well-known application with a broad user base commonly used to improve and assess empirical protein architectures and structure projection and analysis. Protein structural investigation is often a demanding and laborious process [42]. In addition, the ProSA-web calculated the Z-score as -6.53.

### Active sites determination of the protein

**Fig. 6.**
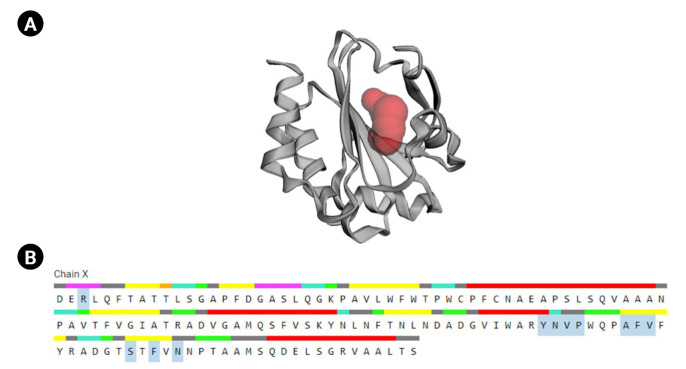
Active site determination. (A) Active sites of the protein. The “red sphere” indicates the active sites of the protein. (B) The amino acid residues in the active site (blue color).

CASTp is a database platform capable of locating zones on proteins, outlining their outlines, determining the dimensions of the regions, and calculating their area. This incorporates pockets on the surface of proteins and hidden vacuums within proteins. The computation includes a pocket and volume spectrum or vacuum, which are mathematically calculated by a solvent-accessible surface (Richards surface) and molecular surface model (surface of Connolly). CASTp could be used to study surface characteristics and protein functioning regions. CASTp delivers a graphical, user-interface-flexible, dynamic display and on-the-fly assessment of user-submitted constructions [43]. The CASTp v.3.0 program demonstrated 11 different active sites in the protein. The highest functioning zones of the modeled protein were recognized between the area of 80.526 and the volume of 37.099 (Fig. 6).

### Antigenicity, allergenicity, and toxicity

Vaccine development in the post-genomic age often commences with the *in silico* assessment of genome data, with the most likely defensive antigens anticipated instead of the cultivation of pathogenic bacteria. Despite the apparent benefits of this method, such as speed and cost-effectiveness, its success is contingent on the precision of antigen prediction. Antigens are identified using sequence alignment in most cases [89-93]. This situation is hazardous for several reasons. Specific proteins may share comparable structures and biological activities despite visible sequence similarity. The antigenicity of a sequence may be encoded in a subtle and convoluted manner, making straightforward detection by sequence alignment impossible [94-96]. Considering the protein’s physical and chemical attributes, the VaxiJen program projected that it was antigenic, with the baseline threshold of 0.4 used as the antigenicity parameter. The overall anticipated antigenicity score was measured as 0.5936.

Allergy overreaches the immune function to a formerly exposed, normally innocuous chemical, leading to skin rash, mucous membrane swelling, sneezing or wheezing, or other aberrant symptoms. The rising prevalence of altered proteins in food, commercial items, laundry detergent, medical therapies, and diagnostics renders anticipating and detecting possible allergies a significant social concern. Using bioinformatics, allergen prediction has been extensively studied, and several tools have been created over the past decade; many are accessible on the complimentary internet [97,98]. Furthermore, the AllerTOP (v. 2.0) anticipated the protein as of probable non-allergen protein. Over the last several decades, scientific study has focused on developing peptide/protein-based treatments for various ailments. With various benefits over small molecules, including high selectivity, significant penetration, and simplicity of production, peptides have emerged as prospective therapeutic agents against various disorders. However, the toxicity of peptide- and protein-based therapies is one of their limitations. To forecast the toxicity of peptides and proteins, we built in silico models in this work [99-101]. The ToxinPred program predicted the protein as nontoxic.

Adaptation between pathogens and their innholders has resulted in several metabolic strategies employed by intracellular infections to cope with the defense responses and nutritional insufficiencies throughout infection. Comprehending how proteins act is essential for explaining how they operate, and this protein contains disulfide oxidoreductase, a crucial enzyme associated with the oxidation of dithiol and/or the reduction of disulfide in target sites. This study reveals the fundamental characteristics of the protein of MTB. Moreover, the protein-protein interactions, active amino acid residues, allergenicity, antigenicity, and toxicity uncover the protein potentiality of MTB infection.

## Figures and Tables

**Table 1.** Protein retrieval

Protein individuality	Protein information
Locus	OHO19689
Amino acid	173 aa
Definition	Hypothetical protein BBW91_12415 [*Mycobacterium tuberculosis*]
Accession	OHO19689
Version	OHO19689.1
GenBank ID	OHO19689.1
Source	*Mycobacterium tuberculosis* (*Mycobacterium tuberculosis* variant tuberculosis)
Organism	*Mycobacterium tuberculosis*
FASTA sequence	>OHO19689.1 hypothetical protein BBW91_12415 [*Mycobacterium tuberculosis*]
	MSLRLVSPIKAFADGIVAVAIAVVLMFGLANTPRAVAADERLQFTATTLSGAPFDGASLQGKPAVLWFWTPWCPFCNAEAPSLSQVAAANPAVTFVGIATRADVGAMQSFVSKYNLNFTNLN ADGVIWARYNVPWQPAFVFYRADGTSTFVNNPTAAMSQDELSGRVAALTS

**Table 2.** Physicochemical parameters

Parameter	Value
Molecular weight	18,382.98
Formula	C_835_H_1274_N_218_O_239_S_6_
Theoretical pI	5.19, 4.98^a^
Total number of atoms	2,572
Total number of positively charged residues (Arg + Lys)	10
Total number of negatively charged residues (Asp + Glu)	11
The estimated half-life	a) 30 h (mammalian reticulocytes, *in vitro*)
	b) >20 h (yeast, *in vivo*)
	c) >10 h (*Escherichia coli*, *in vivo*)
Aliphatic index	86.42
Instability index (II)	29.40
Grand average of hydropathicity (GRAVY)	0.334

a pI calculated by the SMS v.2.0.

**Table 3.** Subcellular localization and protein topology analysis results

Analysis	Result
CELLO (v2.5)	Extracellular
PSORTb (v3.0)	Extracellular
HMMTOP (v.2.0)	1 Transmembrane helix
TMHMM (v.2.0)	1 Transmembrane helix
